# The role of macrophages in liver fibrosis: composition, heterogeneity, and therapeutic strategies

**DOI:** 10.3389/fimmu.2024.1494250

**Published:** 2024-11-20

**Authors:** Xiaocao Ma, Jia Qiu, Shubiao Zou, Liling Tan, Tingting Miao

**Affiliations:** ^1^ Department of Nuclear Medicine, The Second Affiliated Hospital, Jiangxi Medical College, Nanchang University, Nanchang, China; ^2^ Jiangxi Province Key Laboratory of Immunology and Inflammation, Jiangxi Provincial Clinical Research Center for Laboratory Medicine, Department of Clinical Laboratory, The Second Affiliated Hospital, Jiangxi Medical College, Nanchang University, Nanchang, Jiangxi, China; ^3^ Department of Radiology, The Second Affiliated Hospital, Jiangxi Medical College, Nanchang University, Nanchang, China; ^4^ Intelligent Medical Imaging of Jiangxi Key Laboratory, Nanchang, China

**Keywords:** liver fibrosis, ECM, macrophage, heterogeneity, therapeutic strategies

## Abstract

Macrophages, the predominant immune cells in the liver, are essential for maintaining hepatic homeostasis and responding to liver injury caused by external stressors. The hepatic macrophage population is highly heterogeneous and plastic, mainly comprised of hepatic resident kuffer cells (KCs), monocyte-derived macrophages (MoMφs), lipid-associated macrophages (LAMs), and liver capsular macrophages (LCMs). KCs, a population of resident macrophages, are localized in the liver and can self-renew through *in situ* proliferation. However, MoMφs in the liver are recruited from the periphery circulation. LAMs are a self-renewing subgroup of liver macrophages near the bile duct. While LCMs are located in the liver capsule and derived from peripheral monocytes. LAMs and LCMs are also involved in liver damage induced by various factors. Hepatic macrophages exhibit distinct phenotypes and functions depending on the specific microenvironment in the liver. KCs are critical for initiating inflammatory responses after sensing tissue damage, while the MoMφs infiltrated in the liver are implicated in both the progression and resolution of chronic hepatic inflammation and fibrosis. The regulatory function of liver macrophages in hepatic fibrosis has attracted significant interest in current research. Numerous literatures have documented that the MoMφs in the liver have a dual impact on the progression and resolution of liver fibrosis. The MoMφs in the liver can be categorized into two subtypes based on their Ly-6C expression level: inflammatory macrophages with high Ly-6C expression (referred to as Ly-6C^hi^ subgroup macrophages) and reparative macrophages with low Ly-6C expression (referred to as Ly-6C^lo^ subgroup macrophages). Ly-6C^hi^ subgroup macrophages are conducive to the occurrence and progression of liver fibrosis, while Ly-6C^lo^ subgroup macrophages are associated with the degradation of extracellular matrix (ECM) and regression of liver fibrosis. Given this, liver macrophages play a pivotal role in the occurrence, progression, and regression of liver fibrosis. Based on these studies, treatment therapies targeting liver macrophages are also being studied gradually. This review aims to summarize researches on the composition and origin of liver macrophages, the macrophage heterogeneity in the progression and regression of liver fibrosis, and anti-fibrosis therapeutic strategies targeting macrophages in the liver.

## 1 Introduction

Chronic liver disease arises from exposure to diverse pathogenic factors for the long term in the liver, which largely affects the normal physiological functions of the liver. At present, chronic liver disease is a serious public health issue, with approximately 2 million deaths per year on a global scale (1, 2). The reparative response of the liver following inflammatory insults, induced by a variety of exogenous factors (e.g. infection, alcohol consumption, high-fat diet, etc.) or endogenous factors (e.g. reactive oxygen species (ROS), inflammasome activation, etc.), frequently leads to hepatic fibrosis (3). Liver fibrosis is primarily characterized by excessive deposition of ECM components, notably collagen fibers and some other fibrins, such as elastin, in the liver sinusoidal space (4). Prolonged and severe liver fibrosis can trigger liver scar formation via ECM accumulation, and ultimately further progressing into liver cirrhosis, liver cancer, and even liver failure (3, 5). According to previous studies, liver injury caused by various etiologies like chronic viral hepatitis and nonalcoholic fatty liver disease (NAFLD) can progress into liver fibrosis. However, early liver fibrosis can be reversed through effective anti-viral therapy and lifestyle adjustments, indicating that liver fibrosis is a reversible dynamic balance process (6–9). Therefore, elucidating the pathogenesis of liver fibrosis and developing targeted therapeutic drugs hold significant clinical importance and research value.

The progression of liver fibrosis is always in a dynamic balance between fibrogenesis and fibrolysis (10). Hepatic stellate cells (HSCs) are responsible for the secretion of ECM components, specifically collagen, and play a crucial role in the pathogenesis of liver fibrosis. Additionally, macrophages also play a pivotal role in the initiation and progression of liver fibrosis (11). Macrophages are widely distributed in multiple organs and tissues in the body, they can be divided into resident macrophages and monocyte-derived macrophages within the liver sinusoids (12, 13). Interestingly, liver macrophages are a population of immune cells characterized by highly heterogeneous and plastic, and these macrophages could exhibit distinct cellular phenotypes depending on microenvironmental signals (14). Liver macrophages play crucial roles in a range of physiological and pathological processes, including the maintenance of tissue homeostasis, defense against pathogens, and tissue repairment (15).

In recent years, a substantial amount of literature has reported that macrophages claimed great importance in hepatic inflammation and fibrosis, and macrophage infiltration is a common feature of liver fibrosis caused by multiple chronic liver injuries (16). In addition, macrophages exhibit a dual functionality in the progression and regression phases of liver fibrosis due to high cell heterogeneity (17). Therefore, it is urgent to explore the heterogeneity of macrophages in different disease states and develop approaches for inhibiting or reversing liver fibrosis, which not only enhances our comprehension of hepatic macrophages from an immunological perspective, but also contributes to developing effective drugs to treat liver fibrosis targeting macrophage in future studies. This review aims to conclude the composition and origin of hepatic macrophages, as well as the heterogeneity of macrophages in the progression of liver fibrosis. Subsequently, potential anti-fibrotic strategies targeting macrophages are discussed. This data provides a basis for further studying the mechanism of macrophage regulation and developing therapies for liver fibrosis through regulating macrophage heterogeneity.

## 2 The composition and origin of liver Macrophages

Macrophages account for the largest proportion in the liver among all solid organs in the body. Macrophages are crucial in maintaining immune homeostasis, disease progression and outcome (18). According to recent literatures, macrophages are characterized by a high degree of heterogeneity (19). In the past, macrophages were simply classified into pro-inflammatory M1-like macrophages and anti-inflammatory M2-like macrophages. However, this traditional classification method fails to fully display the functional heterogeneity of liver macrophages (19, 20). In the past few years, liver macrophages have been more accurately categorized as KCs, MoMφs, LAMs, and LCMs based on their origin, function and associated surface markers (21). As for mouse KCs, apart from F4/80^hi^CD11b^int^, Clec4F(C-type lectin domain family 4 member F)and Tim-4(T cell immunoglobulin and mucin domain-containing protein-4) are used as specific cell-surface marker. And mouse KCs are characterized by F4/80^hi^CD11b^int^Clec4F^+^Tim4^+^, whereas mouse MoMφs are identified by F4/80^int^CD11b^hi^Clec4F^-^Tim4^-^ (22, 23). Furthermore, Clec4F was identified as a specific marker of resident KCs, but it is expressed relatively late in the development of KCs, making it difficult to identify cells that are developing into KCs (24). Clec2, encoded by the Clec1b gene, is an early marker for KCs and continues to be expressed throughout their lifespan. Therefore, Clec2 is useful for identifying monocyte-derived KCs before they express Clec4F (24, 25). Therefore, KCs and MoMφs in the mouse liver can be effectively distinguished based on their surface marker profiles (26, 27). Human liver macrophages are mainly composed of KCs and MoMϕs, but the macrophage markers are different from those in mice. Human KCs are usually characterized as CD14^+^CD68^+^, especially CD68, a recognized marker of human KCs (27, 28). While human MoMϕs are usually recognized as CD14^+^CCR2^+^ (17). In addition, the liver also contains LAMs and LCMs (24, 29). The surface markers of human LAMs are CD14, CD9, TREM2, and GPNMB (30, 31). No definite surface markers have been identified in the mouse LAMs (24). While LCMs express general macrophage markers such as F4/80, CD64, and CX3CR1 (24, 29).

**Figure 1:**
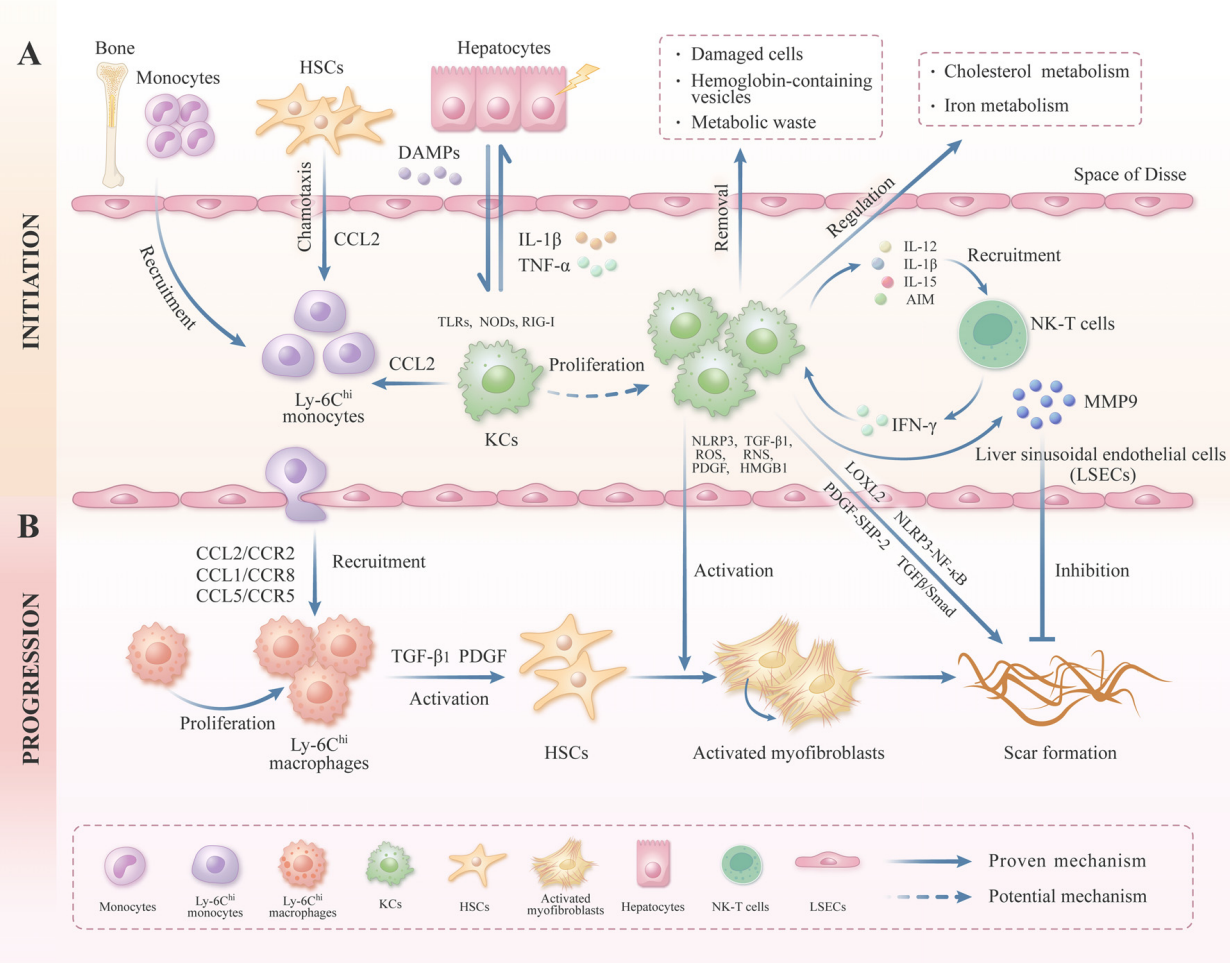
The role of liver macrophage in the initiation and progression of liver fibrosis. This figure provides an overview of the heterogeneity and plasticity of liver macrophages. **(A)** KCs, located in the hepatic sinus endothelium, can be activated by DAMPs after suffering from liver injury, and activated KCs can release macrophage chemokine CCL2 to recruit the Ly6C^hi^ subgroup monocytes to the liver. KCs express a variety of PRRs, including TLRs, NODs, and RIG-I, which facilitate the effective identification and removal of foreign pathogens. KCs can also secrete inflammatory cytokines (e.g.IL-1β and TNF-α) to aggravate the damage of hepatocytes. KCs can secrete IL-12, IL-15, IL-1β, and AIM, which further recruit and activate NK-T cells. In turn, NK-T cells can produce inflammatory cytokines, including IFN-γ, thereby modulating the function of KCs and impacting the progression of liver fibrosis. In addition, KCs can regulate a variety of metabolic pathways, including the cholesterol and iron metabolism. Besides, KCs can remove damaged cells and metabolic waste to maintain the metabolic balance. **(B)** Under the chemotaxis axis, Ly-6C^hi^ subgroup monocytes can be recruited to the liver, where they differentiate into proliferating Ly-6C^hi^ subgroup macrophages, and these macrophages can secrete TGF-β_1_ and PDGF to activate HSCs. In addition, KCs can also release NLRP3, TGFβ, PDGF, ROS, and RNS, then induce the quiescent HSCs activated into myofibroblasts, ultimately secreting ECM and inducing liver fibrosis. KCs can also secrete MMP9, which is conducive to the degradation of liver ECM. In addition, KCs can promote collagen cross-linking and scar formation by modulating the expression of LOXL2.

Under liver homeostatic conditions, resident KCs which originated from fetal yolk sac erythromyeloid progenitors and are mainly located in the liver sinusoids, constitute the primary macrophage population, and the lifespan of KCs in the liver is relatively brief (32). KCs can sustain their population through continuous self-renewal (19). KCs are highly efficient phagocytes that constitute the first line of host defense against pathogens, thereby maintaining hepatic homeostasis (33). In addition, KCs express a variety of pattern recognition receptors (PRRs), including Toll-like receptors (TLRs), nucleotide oligomerization domain-like receptors (NODs), and retinol-inducible gene I (RIG-I), which contributes to the effective identification and elimination of foreign pathogens (17, 34) (**Figure 1**). Simultaneously, KCs are involved in many metabolic pathways, including removing damaged cells, erythrocyte-derived hemoglobin-containing vesicles, and metabolic waste products through scavenger receptors (35, 36). Moreover, KCs can regulate cholesterol metabolism and contribute to maintaining cholesterol homeostasis in the body (37). Meanwhile, KCs can also recycle iron by inducing phagocytosis of damaged erythrocytes, which can participate in maintaining the iron metabolism balance (38). Additionally, KCs play a significant role in mediating immune tolerance (39). Interestingly, KCs can not only act as guardians to maintain liver homeostasis, but also release damage-associated molecular pattern (DAMP)after sensing liver injury, and the DAMP can activate KCs to secrete TNF-α and IL-1β, further causing damage to hepatocytes (40, 41) (**Figure 1**). The number of KCs and monocyte-derived macrophages maintains a dynamic balance in the liver, when the KCs are exhausted or depleted due to various internal or external factors, myeloid-derived monocytes will replenish self-renewing KCs to respond to liver damage (42).

**Figure 2:**
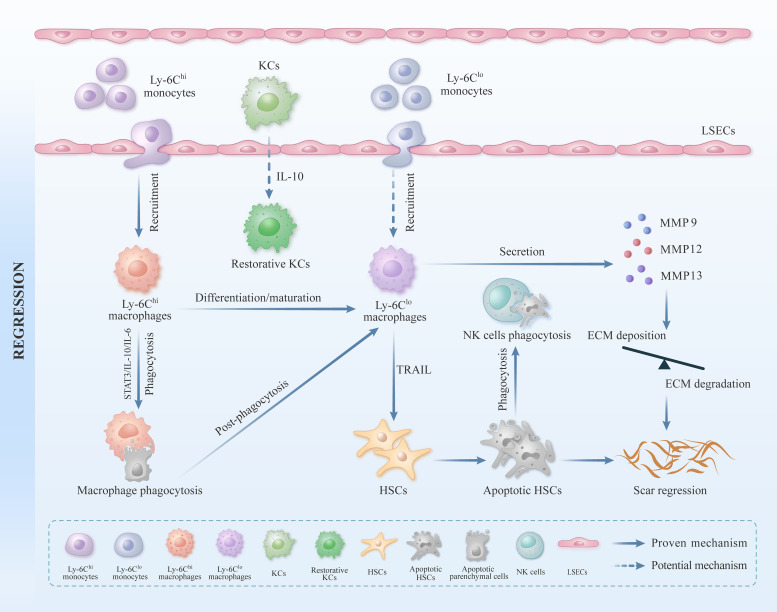
The role of liver macrophages in liver fibrosis repair. Apart from KCs, MoMφs play a crucial regulatory role in liver inflammation and fibrosis. When the liver suffers from external damage, a substantial influx of peripheral monocytes can be recruited to the liver and subsequently differentiate into mature Ly-6C^hi^ subgroup macrophages. These Ly-6C^hi^ subgroup macrophages exhibit pro-inflammatory and pro-fibrotic characteristics. In the stage of liver injury repair, Ly-6C^hi^ subgroup macrophages can be transformed into Ly-6C^lo^ subgroup macrophages by activating STAT3/IL-10/IL-6 signaling pathway, and then engulfing hepatocyte fragments. Ly-6C^lo^ subgroup macrophages can promote ECM degradation by secreting MMP9, MMP12, and MMP13. Ly-6C^lo^ subgroup macrophages can also induce the apoptosis of activated HSCs by expressing TRAIL, thus inhibiting the formation of liver collagen and accelerating the repair process of liver fibrosis.

Compared with KCs, MoMφs derived primarily from peripherally circulating monocytes, constitute only a small percentage of the macrophage pool in healthy livers (43). When the liver was damaged, monocytes derived from the peripheral circulation are quickly recruited to the liver via the CCL2/CCR2 (CC chemokine ligand-2/CC chemokine receptor-2), CCL5/CCR5, and CCL1/CCR8 chemotactic axes, and then trans-differentiated into mature macrophages (17, 44–46) (**Figure 1**). The MoMφs, after being continuously recruited to the liver, dominate the macrophage population and play a crucial role in regulating liver damage and repair (17). Interestingly, MoMφs are highly heterogeneous, composed of a spectrum of functional plastic cells, these MoMφs can undergo constant variation under a dynamically changing micro-environment in the liver (14). MoMφs can be further classified into pro-inflammatory Ly-6C^hi^ subgroup macrophages and anti-inflammatory Ly-6C^lo^ subgroup macrophages according to the surface markers and functional characteristics, the former exhibits pro-inflammatory and pro-fibrotic properties, and the latter displays anti-inflammatory and anti-fibrotic properties (17, 47). Under specific conditions, pro-inflammatory Ly-6C^hi^ subgroup macrophages can trans-differentiate into reparative Ly-6C^lo^ subgroup macrophages via phagocytosis (48) (**Figure 2**). It has been reported that a large number of Ly-6C^hi^ subgroup macrophages can infiltrate into the liver and exert pro-inflammatory and pro-fibrotic functions during the progression stage of carbon tetrachloride (CCL_4_)-induced liver fibrosis, and Ly-6C^hi^ subgroup macrophages can transform into Ly-6C^lo^ subgroup macrophages after CCL_4_ removal, leading to collagen degradation and gradual liver repair (26). Therefore, the MoMφs in the liver are highly plastic and heterogenous cells.

**Figure 3:**
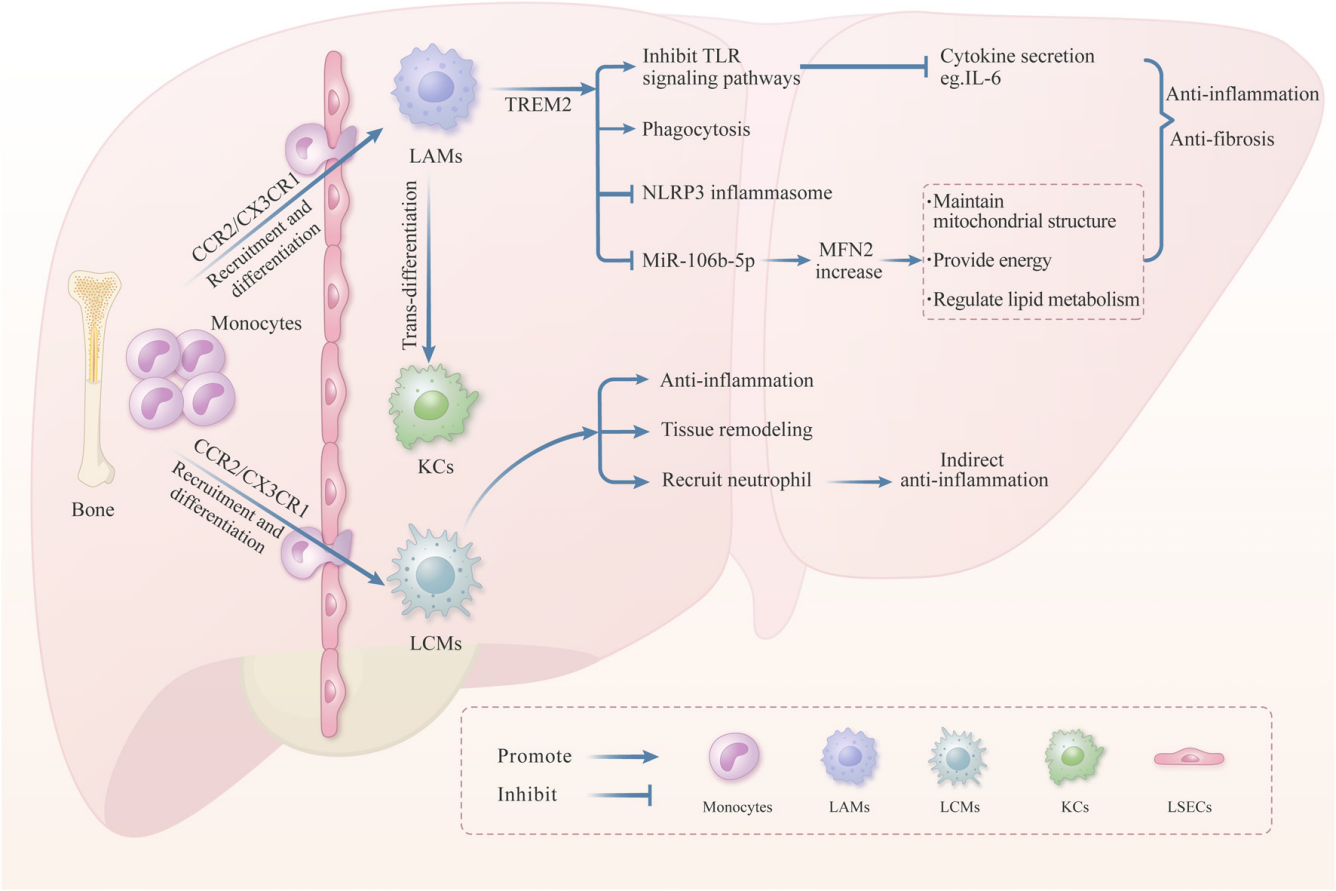
The role of LAMs and LCMs in liver injury. In liver pathological conditions, LAMs precursor cells are recruited to the liver via chemotaxis and then differentiate into LAMs. The transmembrane receptor TREM2, on the surface of LAMs, plays a vital role in regulating the LAMs function. Numerous literatures have reported that TREM2 can promote anti-inflammation and anti-fibrosis through inhibiting the TLRs signaling pathway, promoting phagocytosis, and inhibiting the NLRP3 inflammasome. Moreover, TREM2 can inhibit the production of MiR-106b-5p in the exosomes derived from macrophage, thereby enhancing the MFN2 expression to maintain mitochondrial structure and lipid metabolism balance. Besides, LAMs can transform into KCs when the KCs were exhausted. LCMs located in the liver capsule also play a significant role in liver inflammation and tissue remodeling. LAMs are recruited from peripheral monocytes, and mitigate the dissemination of intraperitoneal bacteria by recruiting neutrophils in the liver, thereby inhibiting the liver inflammation indirectly.

Furthermore, LAMs and LCMs are also two critical subgroups macrophages in the liver. LAMs are mainly situated near the hepatobiliary ducts, and their quantity is relatively low under physiological conditions. In contrast, the population of LAMs increases and predominantly accumulates in adipose and inflammatory tissues in pathological conditions like liver injury (24, 49). LAMs originate from precursor cells derived from peripheral monocytes and possess the capacity for self-renewal. The precursor cells of LAMs express CCR2 and CX3CR1, which facilitate the migration and recruitment of monocytes (50). Research has reported that when KCs are exhausted, LAMs can transform into KCs to replenish the KCs pool in the liver (25). LAMs possess an abundance of intracellular lipid structures and lysosomes, which are indispensable for lipid metabolism, ECM remodeling, and the clearance of apoptotic hepatocytes (49, 51) (**Figure 3**). Numerous studies have investigated the role of LAMs in liver inflammatory injury and tissue remodeling. It was reported that LAMs deficiency in the liver can aggravate liver inflammation and fibrosis induced by nonalcoholic steatohepatitis (NASH) in mice, indicating that LAMs can inhibit the progression of inflammation and liver fibrosis (49, 52). As for LCMs, they originate from peripheral circulating monocytes via CCR2 and CX3CR1 and have elongated dendrites on their cell membrane surface. These dendritic structures facilitate intercellular cross-linking on the liver capsule surface, forming an intricate cellular network. This network plays a crucial role in immune surveillance by sensing and limiting the dissemination of bacteria in the liver capsule (24, 53). In addition, LCMs also contribute to regulating liver inflammation (54) (**Figure 3**).

Human monocytes in the peripheral can also be categorized into distinct subsets with unique phenotypic and functional characteristics (55). The literature indicates that human peripheral blood monocytes can be categorized into classical monocytes (identified as CD14^++^CD16^-^ or CD14^+^CD16^-^), intermediate monocytes (identified as CD14^++^CD16^+^ or CD14^+^CD16^+^), and non-classical monocytes (identified as CD14^-^CD16^++^ or CD14^-^CD16^+^) based on the expression of surface marker (55, 56). To a certain extent, the gene expression profiles of CD14^++^CD16^-^ monocytes and CD14^+^CD16^++^ monocytes in human peripheral blood respectively exhibit similarities to Ly-6C^hi^ subgroup monocytes and Ly-6C^lo^ subgroup monocytes in mouse peripheral blood in previous studies (56, 57). Based on the above literature, the phenotype and function of macrophages are modulated by specific liver micro-environment, ultimately determining whether they exacerbate or ameliorate liver damage. These findings explain why liver macrophages perform different and sometimes even opposite functions during hepatic diseases (58).

## 3 The function of KCs in liver fibrosis

KCs, situated in the periportal area of the hepatic sinusoids, constitute an important component of the innate immune system within the body. KCs maintain their population mainly through proliferation and act as scavengers to maintain hepatic homeostasis (17). Monitoring infectious and non-infectious insults in the liver is a pivotal role of KCs (59, 60). KCs can modulate the occurrence and progression of liver fibrosis by regulating NOD-like receptor protein 3 (NLRP3) inflammasome, PRR, transforming growth factor-β (TGF-β) signaling pathway, and platelet-derived growth factor (PDGF) signaling pathway (61–64) (**Figure 1**). For example, the KCs-derived NLRP3 inflammasome can promote the occurrence of liver fibrosis by activating the nuclear factor kappa-B (NF-κB) signal pathway in *Schistosoma japonicum*-infected mice (61). KCs can also release pro-inflammatory cytokines, including TNF-α and IL-1β, and recruit peripheral mononuclear macrophages to the liver through the CCL2/CCR2 axis, further aggravating liver fibrosis (17). In addition, KCs can also stimulate quiescent HSCs transformed into activated HSCs through various mechanisms, leading to increased production and secretion of ECM (65, 66). For example, TGF-β, a pro-fibrotic cytokine secreted by KCs, can activate HSCs by activating the TGF-β/Smad signaling pathway, further promoting ECM deposition (67, 68). PDGF, a potent proliferative cytokine secreted by KCs, can induce tyrosine phosphorylation and activate the downstream signaling molecule SHP-2 (Src homology region 2 domain-containing protein tyrosine phosphatase-2) by binding to the cell-surface receptors on HSCs, and then exacerbate liver fibrosis in mice (69–71). It is well established that the activated KCs can also produce ROS/reactive nitrogen species (RNS) to stimulate the activation and proliferation of HSCs, ultimately increasing ECM deposition in the liver (66). Furthermore, it has been demonstrated that HMGB1 (High-mobility group box-1), produced by hepatic cells like KCs and hepatocytes, can activate HSCs to express collagen I via its receptor RAGE (the Receptor for Advanced Glycation End-products), thereby exacerbating liver inflammation and fibrosis (72). Some studies have further confirmed that KCs depletion can not only inhibit the production of IL-1β and TNF-α but also inhibit the activation of HSC, thereby alleviating liver fibrosis in a bile duct ligation (BDL)-induced liver fibrosis mouse model (73). Besides, when viruses like hepatitis B (HBV) infect the liver, KCs can detect the danger signals and recruit circulating monocytes to the liver, where these monocytes differentiate into MoMφs, ultimately regulating anti-viral immunity. Furthermore, these macrophages can also release TGF-β_1_ to promote the progression of viral hepatitis to liver fibrosis (74, 75). In addition, KCs can also directly regulate ECM remodeling and exhibit significant cellular heterogeneity, such as KCs can promote collagen cross-linking and scar formation by modulating the expression of lysyl oxidase-like protein 2 (LOXL2). Conversely, KCs can also secret matrix metalloproteinase (MMP), such as MMP9, to degrade collagen under certain circumstances (32).

KCs can regulate the development and progression of liver fibrosis through the mechanisms above. Additionally, KCs can also interact with various immune cells such as natural killer T (NK-T)cells, consequently impacting the development of liver fibrosis (**Figure 1**). KCs can secrete IL-12, IL-15, IL-1β, and apoptosis inhibitors of macrophage (AIM) to recruit and activate NK-T cells. In response, NK-T cells can produce pro-inflammatory cytokines such as interferon-gamma (IFN-γ) to modulate the function of KCs, influencing the progression of liver fibrosis (76) (**Figure 1**). Furthermore, KCs can release chemical mediators to recruit neutrophils to the liver; whereupon neutrophils can release inflammatory mediators such as ROS to participate in liver repair (77, 78). In conclusion, KCs play a significant role in the regulation of liver fibrosis through various direct and indirect mechanisms. Nevertheless, further research is needed to determine whether KCs can be converted into reparative KCs and enhance the hepatic regenerative response during liver injury repair (**Figure 2**).

## 4 The role of MoMφs in liver fibrosis

Except for KCs, MoMφs are also essential regulatory cells for liver inflammation and fibrosis. When the liver suffers from injury, KCs are capable of sensing liver damage and releasing inflammatory cytokines, such as TNF-α and IL-1β. Simultaneously, a large number of peripherally derived monocytes can recruit to the liver and differentiate into mature MoMφs (17) (**Figure 1**). In recent years, MoMφs have drawn significant attention due to their high heterogeneity and plasticity. In the initial phase of chronic persistent liver fibrosis, MoMφs are predominant in the liver, and they can activate HSCs by secreting TGF-β_1_ and PDGF, which further exacerbates liver fibrosis (79, 80) (**Figure 1**). During the regression phase of liver fibrosis, MoMφs can participate in ECM degradation by secreting MMPs such as MMP9, MMP12, and MMP13, which facilitates the regression of liver fibrosis (81) (**Figure 2**). MoMφs can exhibit different cell phenotypes based on the local environmental cue to maintain liver homeostasis or affect disease progression (82). For example, macrophages can secrete cytokines, chemokines, and ROS to modulate liver inflammation and fibrosis in both alcoholic liver disease (ALD) and NAFLD (83, 84). In ALD, ethanol and its metabolite acetaldehyde can activate KCs and then lead to the release of pro-inflammatory mediators such as IL-1β and TNF-α, which results in an acute pro-inflammatory response, simultaneously accompanied by a small number of MoMφs infiltration (21). In NAFLD, the chronic low-grade inflammation induced by lipid accumulation and metabolic dysregulation can lead to substantial MoMφs infiltration in the liver. In the initial stage of NAFLD, macrophages display a pro-inflammatory and phagocytic phenotype that facilitates the clearance of lipid-overloaded hepatocytes. Conversely, in the advanced stages of NAFLD, there is a predominance of M2 macrophages characterized by an anti-inflammatory phenotype. Consequently, pathogenic and reparative macrophages within the liver microenvironment undergo dynamic regulation in NAFLD (84).

Numerous studies have indicated that the conventional M1/M2 binary classification method, which distinguishes between classically activated and alternatively activated macrophages, this classification is insufficient to capture the full complexity of macrophages (19, 85). In recent years, more researchers have categorized MoMφs into pro-inflammatory Ly-6C^hi^ subgroup macrophages and reparative Ly-6C^lo^ subgroup macrophages based on the expression of the cell surface glycoprotein Ly-6C (21). In the context of liver injury, pro-inflammatory Ly-6C^hi^ subgroup macrophages are primarily recruited to the liver through chemokines, including CCL1, CCL2, and CX3CL1, subsequently exacerbating liver inflammation and fibrosis (21, 47) (**Figure 1**). Previous studies have indicated that selective depletion of hepatic Ly-6C^hi^ subgroup macrophages can impede HSCs activation and reduce ECM deposition during the initial stage of liver fibrosis, while selective depletion of hepatic Ly-6C^lo^ subgroup macrophages will be detrimental in the repair phase of the liver fibrosis (26, 86). Baeck. C et al. demonstrated that the administration of the CCL2 inhibitor mNOX-E36 could effectively impede the recruitment of Ly-6C^hi^ subgroup macrophages to the liver, thereby reducing the degree of liver fibrosis. The above findings indicate that Ly-6C^hi^ subgroup macrophages predominantly exert a pro-inflammatory and pro-fibrotic effect (87). During the repair stage of liver injury, it has been observed that Ly-6C^hi^ subgroup macrophages can transform into Ly-6C^lo^ subgroup macrophages under specific circumstances *in vivo*. *In vitro* studies have demonstrated that this process was mainly accomplished by engulfing apoptotic hepatocyte fragments. Recent research has further indicated that the activation of the STAT3/IL-10/IL-6 signaling pathway plays a crucial role in mediating phagocytosis and orchestrating the phenotypic conversion of these two subgroups of macrophage (88, 89) (**Figure 2**).

In addition, the phenotypic switch from Ly-6C^hi^ subgroup macrophages to Ly-6C^lo^ subgroup macrophages is influenced by the interaction between macrophages and other immune cells. For instance, Ly-6C^hi^ subgroup macrophages can promote neutrophils generating ROS and promote CD4^+^T cells generating IL-4. These processes can facilitate the phenotype switch of two subgroups of macrophages (90–92). The phenotypic conversion of Ly-6C^hi^ subgroup macrophages to Ly-6C^lo^ subgroup macrophages plays a significant role in facilitating the regression of liver fibrosis induced by multiple factors in mice (93). Additionally, the phenotypic transformation of two subgroups of macrophages in the liver marks the shift from the pro-inflammation initiation phase to the anti-inflammatory and resolution phase (94, 95). In summary, the phenotypic transformation of liver macrophage subsets plays a crucial role in the reversal of liver fibrosis. Further research is warranted to determine whether Ly-6C^lo^ subgroup monocytes in peripheral blood can be recruited to the liver during the repair phase of liver injury, where they may differentiate and mature into Ly-6C^lo^ subgroup macrophages, thereby potentially facilitating the resolution of liver fibrosis (**Figure 2**).

Previous studies demonstrated that collagen degradation was weakened when Ly-6C^lo^ subgroup macrophages were knocked out in the repair stage of the CCL_4_-induced liver fibrosis mouse model, which strongly proves that Ly-6C^lo^ subgroup macrophages play a crucial role in degrading liver collagen (26). Ly-6C^lo^ subgroup macrophages primarily repair liver fibrosis by upregulating MMPs (MMP9, MMP12, and MMP13), which facilitates the degradation of ECM. These cells can also increase the expression of some growth factors like hepatocyte growth factor and insulin-like growth factor for hepatocyte repairment. Furthermore, Ly-6C^lo^ subgroup macrophages can upregulate phagocytosis-related genes like MARCO (macrophage receptor with collagenous structure), which in turn fuel themselves to engulf apoptotic cells (26, 88, 91, 95–97). It has been reported that the activation of HSCs played a pivotal role in the occurrence and development of liver fibrosis (3). Ly-6C^lo^ subgroup macrophages can induce the apoptosis of activated HSCs through expressing TRAIL (TNF-related apoptosis-inducing ligand), subsequently rendering them susceptible to cytotoxic NK cells, besides, Ly-6C^lo^ subgroup macrophages can also revert the activated HSCs back to a quiescent state (98, 99) (**Figure 2**). In conclusion, the aforementioned studies have reveal the significant contribution of Ly-6C^lo^ subgroup macrophages in the process of repairing chronic liver fibrosis.

## 5 The role of LAMs and LCMs in liver fibrosis

The number of LAMs is relatively tiny in normal liver. However, during pathological conditions such as hepatic steatosis, liver inflammation, and liver fibrosis, a large number of peripheral monocyte-derived LAMs precursor cells are recruited to the liver by the CCL2/CCR2 axis and then differentiate into LAMs (24, 50). Studies indicated that knocking out CCR2 can lead to an absence of LAMs in the liver, which aggravates liver inflammation and fibrosis induced by NASH in mice. Therefore, LAMs could inhibit the progression of liver inflammation and fibrosis (50). TREM2, a transmembrane receptor of the immunoglobulin superfamily, has been identified on the surface of LAMs and is pivotal for modulating their function (100). TREM2 can not only recognize lipids and apolipoproteins but also combine with phospholipid molecules on the surface of apoptotic cells, thereby regulating processes including lipid metabolism and cellular phagocytosis in the liver (101). Furthermore, TREM2 can also regulate liver inflammation and tissue repair (49). In murine models of NAFLD, exosomes derived from TREM2-deficient macrophages exhibit elevated levels of miR-106b-5p, which leads to the downregulation of mitochondrial fusion protein 2 (MFN2), thereby damaging mitochondrial architecture and accelerating NAFLD (102) (**Figure 3**). Some studies have also indicated that macrophage-specific knockout of TREM2 would impair its phagocytic capacity for apoptotic hepatocytes, further aggravating the progression of NASH (52). Other studies have demonstrated that mice deficient in TREM2 exhibit aggravated hepatic inflammation in liver injury models induced by CCl_4_ and acetaminophen (APAP), and the mechanism is related to enhanced TLR signal pathways and subsequent pro-inflammatory cytokines secretion (103). The latest research shows that TREM2^+^LAMs can inhibit NLRP3 activation and pro-inflammatory cytokine secretion, then degrade the ECM in the liver, thereby accelerating the regression of liver fibrosis (104) (**Figure 3**). The above studies indicate that LAMs are involved in regulating liver lipid metabolism and cellular phagocytosis through their surface receptor TREM2, and play an essential role in inhibiting liver inflammation and facilitating the repair of liver injury (**Figure 3**). Future research may concentrate on further elucidating the functions of LAMs and developing effective therapeutic approaches for liver fibrosis based on the relevant target.

LCMs also play an essential regulatory role in liver injury (**Figure 3**). Research indicates that LCMs can mitigate the dissemination of intraperitoneal bacteria in the liver, which was achieved by recruiting neutrophils in response to bacterial detection, consequently attenuating hepatic inflammation (53). In a murine model of liver injury induced by 5-chloro-2-(2,4-dichlorophenoxy), LCMs exhibited a shift from M1 to M2 macrophage phenotypes, suggesting their potential involvement in regulating liver tissue remodeling and fibrosis by releasing anti-inflammatory or pro-resolving mediators (105). In the advanced stages of the NAFLD mouse model, the number of liver LCMs increased, accompanied by morphological alterations, which suggests that LCMs may have a regulatory function in the progression of advanced-stage NAFLD (54). The literature above indicates that LCMs constitute a significant subpopulation of macrophages involved in the regulation of liver injury (**Figure 3**). However, little evidence addresses the role of LCMs in liver fibrosis, further research is warranted to elucidate the function of LCMs in this context.

## 6 Potential therapeutic strategies to target macrophages for liver fibrosis

### 6.1 The regulation of intestinal microbiota

Increased bacterial ectopy is one of the important signs of chronic liver diseases, as pathogen-associated molecular patterns (PAMPs) can not only cause typical infectious complications but also activate TLRs like TLR4 on macrophages, thereby causing liver inflammation and fibrosis (106, 107). A great number of bacterial products that originate from the hepatic portal vein can activate the TLR4 receptor, further activating HSCs and recruiting peripheral inflammatory macrophages to the liver, ultimately leading to liver fibrosis (21, 106, 108). Some studies have shown that the endotoxins produced by gram-negative bacteria can induce hepatic fibrogenesis. The transfer of fecal microbiota from healthy donors to individuals with chronic liver diseases, known as fecal microbiota transplantation (FMT), has emerged as a potential novel therapeutic approach, which further reinforces the close link between gut microbiota and liver fibrosis (109, 110). Clinical studies have confirmed that intestinal flora imbalance can drive the progression of liver fibrosis by affecting macrophages, while the antibiotic rifaximin has been found to alleviate alcoholic liver fibrosis by modulating intestinal microflora (108, 111). The above studies indicate that rifaximin has the potential to ameliorate liver fibrosis by targeting macrophages. In addition, the combination therapy of vancomycin, gentamicin, and meropenem shows promise in improving intestinal flora and treating liver disease, which is currently being explored in clinical trials(NCT03157388) (112). It has also been reported that supplementation with probiotic *Lacticaseibacillus rhamnosus* can potentially mitigate liver fibrosis by inhibiting bile acid synthesis and enhancing bile acid excretion (113). The above studies support the existence of cross-talk between intestinal flora and liver diseases, specifically liver fibrosis. Consequently, modulating intestinal flora may represent a novel approach for targeting macrophages in the treatment of liver fibrosis or other liver diseases.

### 6.2 The inhibition of IL-1β signaling pathway

Numerous studies have confirmed a correlation between the NLRP3 inflammasome and liver fibrogenesis (130, 131). The studies demonstrated that NLRP3 inflammasome activation can induce the KCs activation and then release inflammatory cytokines such as IL-1β during liver injury, which plays a crucial role in the progression of liver fibrogenesis (61, 131). IL-1β, a potent pro-inflammatory cytokine, exerts a pro-inflammatory role through interaction with the interleukin-1 receptor (IL-1R1) in an autocrine or paracrine manner, thereby driving the occurrence of liver fibrosis (132, 133). It has been shown that a naturally existing IL-1R1 antagonist (IL-1Ra)can block the biological interaction between IL-1β and its cell surface receptors. Anakinra, a specific antagonist of the IL-1β receptor, can ameliorate liver fibrosis caused by various etiological factors (114–116). The aforementioned studies suggest that blocking the IL-1β receptor may be a promising strategy for the treatment of liver diseases.

### 6.3 The inhibition of the chemotactic axis in macrophages

In the progress of liver fibrosis, peripheral pro-inflammatory mononuclear MoMφs can be attracted to the liver depending on CCL2/CCR2, CCL5/CCR5, and CCL1/CCR8 chemokines axis and aggravate liver fibrosis (44, 134). In this context, inhibiting the recruitment of pro-inflammatory MoMφs to the liver by interfering with the chemotactic axis is a significant therapeutic strategy. mNOX-E36, a CCL2 inhibitor, can suppress the recruitment of pro-inflammatory MoMφs to the liver in murine models of CCL_4_-induced liver fibrosis and methionine-choline-deficient diet (MCD)-induced non-alcoholic steatohepatitis, therefore, the balance of macrophage subgroups can shift to a state that was dominated by reparative MoMφs, ultimately contributing to the fibrosis regression (87, 117, 135). In recent years, numerous studies have extensively reported that Cenicriviroc (CVC) functions as a dual antagonist of CCR2/CCR5 (118). The usage of CVC has been shown to effectively block the recruitment of pro-inflammatory mononuclear MoMφs to the liver that is mediated by the CCL2/CCR2 and CCL5/CCR5 axes, which consequently inhibits both hepatic inflammation and fibrosis (136, 137). In a randomized, double-blind, placebo-controlled trial, Friedman et al. found that hepatic inflammation and fibrosis were significantly improved following a one-year treatment with CVC in NASH (non-alcoholic fatty liver disease activity score NAS ≥ 4) or liver fibrosis (stages I-III) patients (119). Furthermore, the anti-fibrotic effect of CVC treatment can be maintained in the second year (120). Besides, propagermanium (a CCR2 inhibitor) and maraviroc (a CCR5 inhibitor) are also considered as relative chemotactic axis inhibitors, which all promote the amelioration of NASH in murine models (121, 122). It has been reported that CCR8 knockout can inhibit the recruitment of MoMφs derived from peripheral monocytes to the fibrotic liver, thereby alleviating experimental liver inflammation and fibrosis induced by either CCL_4_ or BDL in mice (45). Furthermore targeting the CCL1/CCR8 chemotactic axis can also alleviate inflammatory and fibrotic damage in the lung and peritoneum (138, 139). The above data indicates that the CCL1/CCR8 chemotactic axis is important for driving inflammation and fibrosis in liver or other organs. However, no clinical trials have explored whether blocking the CCL1/CCR8 chemotactic axis can inhibit liver inflammation and fibrosis. Therefore, targeting the CCL1/CCR8 chemotactic axis represents a promising potential approach for clinical intervention in liver inflammation and fibrosis.

These studies show that the chemotaxis effects mediated by chemokines and their receptors initiate the recruitment of peripheral mononuclear MoMφs to the damaged liver. Hence, the inhibition of CCL2/CCR2, CCL5/CCR5, and CCL1/CCR8 chemotaxis shows potential efficacy to repair liver fibrosis.

### 6.4 Galectin-3 antagonist

Galectin-3 (Gal-3) is a β-galactoside-binding lectin that can be produced from hepatic macrophages (81, 140). Gal-3 exerts multiple regulatory effects on the innate immune and adaptive inflammatory response (141). It has been reported that Gal-3 is involved in the development of fibrosis in various tissues by influencing phenotypic transformation and migration of macrophages. Moreover, Gal-3 can also function as a pro-inflammatory and pro-fibrotic mediator in the liver (142, 143). Jiang et al. reported that Gal-3-knockout could inhibit the HSCs activation and effectively alleviate liver fibrosis while Gal-3 overexpression could reverse it in mice after BDL (144). In addition, it was also observed that Gal-3 inhibition can also suppress liver inflammation and liver fibrosis in mouse models of NASH (123). The above studies have shown that the Gal-3, secreted by macrophages, could contribute to hepatic fibrogenesis, this notion may suggest that Gal-3 could serve as a potential therapeutic target for the patients with liver fibrosis. As previously reported, GR-MD-02, a Gal-3 inhibitor, is a complex carbohydrate compound extracted from natural plants. In a phase III clinical trial for NASH-induced advanced liver fibrosis, GR-MD-02 has been shown high safety and tolerability on the human body, as well as the potential to ameliorate liver fibrosis (123). GB1211, another Gal-3 inhibitor, also has been confirmed to have favorable safety and tolerability in phase I clinical trial, which indicates that GB1211 warrants further clinical trial research for anti-fibrotic treatments (124). In summary, the above studies suggest that targeted inhibition of macrophage-derived Gal-3 is conducive to the repairment of liver fibrosis.

### 6.5 PPARs agonist

Peroxisome proliferator-activated receptors (PPARs) are a group of key nuclear transcription factors that play important roles in regulating lipid metabolism, cell differentiation, and maintaining liver homeostasis (145). In mammals, PPARs consist of three isoforms: PPAR-α, PPAR-δ (also referred to as PPAR-β), and PPAR-γ (146). The liver is an organ that mainly regulates systemic metabolism and maintains energy homeostasis, with PPARα governing lipid metabolism in the liver, and the dysregulation of lipid metabolism may lead to steatohepatitis or even liver fibrosis (147). PPAR-δ has been shown to inhibit liver steatosis and block the progression of steatohepatitis by upregulating low-density lipoprotein receptor(LDL-R) (148). Additionally, PPARγ can inhibit NF-κB activity through directly binding to its p65 subunit, thereby improving steatohepatitis (149). Research has reported that PPARs activation can inhibit the advancement of liver fibrosis by regulating the inflammatory responses in macrophages. Lanifibranor, a PPARs agonist with broad-spectrum activity, can attenuate the inflammatory response induced by palmitic acid on human monocytes and mouse bone marrow-derived macrophages *in vitro* experiments (125). *In vivo* animal experiments also demonstrated that Lanifibranor has the potential to ameliorate liver inflammation and fibrosis induced by NAFLD (125, 150). In addition, in a phase IIb randomized controlled clinical trial, it has been corroborated that lanifibranor treatment for six months can alleviate liver fibrosis induced by NASH. Building on this evidence, phase III clinical trials are currently being explored (151). The above literature confirmed that modulating the activation of PPARs to control the inflammatory response of macrophages is a promising therapeutic approach for liver inflammation and fibrosis.

### 6.6 The Farnitol X receptor agonist

The farnitol X receptor (FXR) agonist is a nuclear receptor that plays a crucial role in regulating bile acid and lipid homeostasis, enhancing cholesterol transport in macrophages, and serving as a key regulator in hepatic steatosis, inflammation, and fibrosis (152, 153). FXR activation can not only decrease the synthesis of bile acids, but also suppress the production of pro-inflammatory cytokines from hepatic infiltrating inflammatory cells, thereby preventing the progression of liver fibrosis (154). Obeccholic acid, an FXR agonist, is beneficial to lipid metabolism and glucose metabolism, and is considered as a leading candidate for the treatment of liver fibrosis induced by NASH (126). These studies suggest that FXR may be an important therapeutic approach to improve the outcome of liver fibrosis by affecting liver macrophages.

### 6.7 Splenectomy

Advanced liver fibrosis and cirrhosis often have multiple complications, including splenomegaly and hypersplenism (127). It has been reported that advanced liver fibrosis and cirrhosis are often accompanied by thrombocytopenia, which results from the destruction of circulating platelets during secondary portal hypertension or hypersplenism (155). Animal experimental studies have demonstrated that thrombocytopenia can further aggravate liver fibrosis induced by BDL and CCL_4_ in mice. The underlying mechanism involves the upregulation of the pro-fibrotic cytokine TGF-β1 and the downregulation of MMPs in the liver. Consequently, therapies targeting thrombocytopenia may serve as an effective strategy for repairing advanced liver fibrosis and cirrhosis (155, 156). Recent research has indicated that splenectomy is beneficial for liver repair and regeneration. Mechanistically, splenectomy has been shown to alleviate advanced liver fibrosis by ameliorating thrombocytopenia, and splenectomy can also facilitate the recruitment of Ly-6C^hi^ subgroup macrophages into the liver and differentiation into Ly-6C^lo^ subgroup macrophages, thereby accelerating the regression of hepatic fibrosis induced by thioacetamide (TAA) and concanavalin A (ConA) in mice (127, 128). In addition, advanced liver fibrosis and cirrhosis are frequently associated with gut microflora dysbiosis. However, splenectomy can ameliorate liver fibrosis and cirrhosis by restoring gut barrier function and maintaining gut microbiota balance by inhibiting the TLR4/NLRP3 signaling pathway (128, 129). The above reports enhance our understanding of the mechanism by which splenectomy reverses advanced liver fibrosis and cirrhosis. Although splenectomy for treating liver cirrhosis is available in clinical practice, many patients with liver cirrhosis have contraindications for splenectomy. Therefore, it is critical to explore non-surgical alternatives for the treatment of liver fibrosis and cirrhosis.

### 6.8 Other treatment strategies

Except for the above therapies, some other interventions such as macrophage adoptive transfer, and drug-directed delivery approaches can also impact the progress and outcome of liver fibrosis by specifically targeting macrophages. Thomas et al. report that the adoptive transfer of anti-inflammatory macrophages can alleviate CCL_4_-induced liver fibrosis in mice (157). In addition, it was reported that liposomes can be routinely used as carrier materials for efficiently delivering drug molecules to pathological sites. For example, dexamethasone (Dex)-loaded liposomes have the potential to induce anti-inflammatory polarization of hepatic macrophages. In the context of experimental chronic liver damage, the administration of dex-loaded liposomes has been shown to markedly alleviate liver injury and fibrosis (158). Moreover, the phagocytosis function of macrophages can promote the inflammatory macrophages transforming into reparative macrophages in a well-characterized murine model of CCl_4_-induced liver fibrosis, leading to accelerated degradation of liver fibrosis (26). Overall, these studies suggest that the targeted regulation of macrophage activity and function may be a promising approach for treating liver fibrosis.

Liver macrophages play pivotal roles in the liver fibrosis during its initiation, progression, and regression. Therefore, targeting these macrophages is essential for antifibrotic therapeutic strategies. A variety of therapeutic approaches and related mechanisms on the above are summarized and listed in **Table 1**.

**Table 1:** Therapeutic strategies of anti-liver fibrosis targeting macrophages.

Therapies of treatment	Mechanism	Drugs or others	Reference
Antibiotics; FMT	Regulate fecal microbiota and inhibit macrophage activation	Rifaximin; The combination therapy of vancomycin, gentamicin and meropenem	(108–113)
IL-1β receptor antagonist	Inhibit the activation of inflammasome	Anakinra	(114–116)
The inhibition of the chemotactic axis in macrophages	Inhibit monocyte recruitment	mNOX-E36; Cenicriviroc; Maraviroc; Propagermanium	(117) (118–120) (121) (122)
Gal-3 antagonist	Inhibit the activation of inflammatory macrophage	GR-MD-02 GB1211	(123) (124)
PPARs agonist	Regulate the inflammatory responses in macrophages	Lanifibranor	(125)
FXR agonist	Increase cholesterol transport in macrophage, regulate the inflammatory responses in macrophages	Obeccholic acid	(126)
Splenectomy	Inhibit thrombocytopenia, increase Ly-6C^lo^ macrophages number, improve intestinal flora	Splenectomy	(127–129)
Targeted delivery of drug molecules	Promote the anti-inflammatory polarization of liver macrophages	Dex-loaded liposomes	(158)

## 6 Conclusion and perspectives

Liver macrophages play an essential role in regulating hepatic homeostasis, maintaining immune tolerance, and influencing the outcome of liver diseases. Liver macrophages are a highly heterogeneous cell population that composed of KCs, MoMφs, LAMs, and LCMs. These macrophages perform diverse regulatory functions in various liver microenvironments. Under the condition of various liver injury, KCs are capable of modulating the occurrence and development of liver fibrosis through multiple pathways such as inflammasome. In addition, monocytes in the peripheral circulation can be recruited to the injured liver via chemokine receptor-ligand axis and subsequently differentiated into mature macrophages. Latest reports have highlighted the dual functions of hepatic MoMφs in the development and progression of liver fibrosis. In the progressive stage of liver fibrosis, the hepatic MoMφs are predominantly comprised of pro-inflammatory Ly-6C^hi^ subgroup macrophages, which primarily exerts pro-inflammatory and pro-fibrotic function. In the resolution stage of liver fibrosis, the hepatic MoMφs are predominantly composed of anti-inflammatory Ly-6C^lo^ subgroup macrophages, which mainly exhibit both anti-inflammatory and anti-fibrotic properties. Furthermore, LAMs play a crucial role in mitigating liver inflammation and facilitating the repair of liver injury. Similarly, LCMs are also involved in the regulation of liver injury. In recent years, many literatures have extensively documented the heterogeneity of macrophages in the liver under both homeostatic conditions and a variety of disease states. Liver macrophages could be a promising therapeutic target for various liver diseases. Strategies aimed at decreasing the influx of inflammatory monocyte-derived macrophages to the liver and promoting their transition to a reparative phenotype are the therapeutic focus. Several potential treatments targeting liver macrophages are currently undergoing clinical trials. Further investigation of the phenotype and functions of macrophages in different stages and developing effective therapeutic approaches is needed to reverse liver fibrosis.
